# Metropolitan Hospital Sunday Fund

**Published:** 1893-08-05

**Authors:** 


### METROPOLITAN HOSPITAL SUNDAY FUND.

### PARTICULARS OF THE AWARDS FOR 1893.

The following is the report of the Committee of Distribution adopted by the Council on the 1st inst., Sir Sydney Waterlow, Bart., in the chair:—

Your Committee have the honour to recommend the payment of awards to the 181 institutions enumerated in the appendix to this report (see page ), being eight more than last year, and an increase of 76 since the first awards were made in 1873. The total amount available for distribution, after allowing for liabilities and the usual current expenses, is £35,400. Of this total £35,066 is now recommended to 127 hospitals and 54 dispensaries. Five per cent. of the total collected—viz., about £1,900—is set apart to purchase surgical appliances, in obedience to Law XII., in monthly proportions during the ensuing year. Your Committee recommend that all payments to the Fund after August 1st be carried to the credit of next year's Fund.

In compliance with an order of the Council, and for the special use of its members, tables of statistics have been prepared as usual, showing an analysis of the number of beds in hospitals, the cost of patients both at hospitals and dispensaries, the proportionate expense of management, as well as other valuable information, and copies are sent to every member of the Council.

The number of deputations representing committees of various hospitals, &c., invited to confer with your Committee, and to offer explanations on matters of apparently unsatisfactory character, was 10. Of these, eight elected to leave their awards entirely in the hands of this Committee; two deputations attended, and of the ten above mentioned, all are recommended on reduced bases. One application was received from a "Sick Club" under name of a "Dispensary," but was not deemed eligible for an award. Awards are therefore recommended to 171 institutions on full bases, and to 10 institutions on reduced bases.

### Twenty-six General Hospitals.

Charing Cross Hospital, £875; French Hospital, £297 10s.; German Hospital, £533 15s.; Great Northern Central Hospital, £306 5s.; Guy's Hospital, £525; Italian Hospital, £70; King's College Hospital, £1,312 10s.; London Hospital, £2,887 10s.; Metropolitan Hospital, £525; Miller Hospital and Royal Kent Dispensary, £218 15s.; North-West London Hospital, £288 15s.; Poplar Hospital, £218 15s.; Queen's Jubilee Hospital, £52 10s.; Royal Free Hospital, £787 10s.; St. George's Hospital, £1,137 10s.; SS. John and Elizabeth Hospital, £93 5s.; St. Mary's Hospital, £1,837 10s.: Seamen's Hospital Society, £787 10s.; The Middlesex Hospital, £1,662 10s.; Training Hospital, Tottenham, £262 10s.; University College Hospital, £1,093 15s.; West Ham Hospital, £157 10s.; West London Hospital, £525; Westminster Hospital, £918 15s.; London Homœopathic Hospital, £122 10s.; London Temperance Hospital, £498 15s.

### Special Hospitals.

Five Chest Hospitals.—City of London Hospital for Diseases of the Chest, Victoria Park, £805; Hospital for Consumption, Brompton, £1,268 15s.; North London Consumption Hospital, Hampstead, £262 10s.; Royal Hospital for Diseases of the Chest, City Road, £262 10s.; Royal National Hospital for Consumption (Ventnor), £262 10s.

Twelve Children's Hospitals.—Alexandra Hospital for Hip Disease in Childhood, W.C., £[illegible]75; Belgrave Hospital for Children, S.W., £131 5s.; Cheyne Hospital for Incurable Children, S.W., £135 12s. 6d.; East London Hospital for Children, E., £507 10s.; Evelina Hospital for Sick Children, Southwark, S.E., £437 10s.; Home for Incurable Children, Maida Vale, W., £61 5s.; Home for Sick Children, Sydenham, S.E., £122 10s.; Hospital for Sick Children, Great Ormond Street, W.C., £665; North Eastern Hospital for Children Hackney Road, N.E., £331 10s.; Paddington Green Hospital for Children, W., £140; Victoria Hospital for Children, King's Road, Chelsea, S.W., £393 15s.; "The Vine," Sevenoaks," £26 5s.

Six Lying-in Hospitals.—British Lying-in Hospital, Endell Street, W.C., £48 2s. 6d.; City of London Lying-in Hospital, City Road, E.C., £43 15s.; Clapham Maternity Hospital, £17 10s.; East End Mother's Home, £35; General Lying-in Hospital, Lambeth, S.E., £52 10s.; Queen Charlotte's Lying-in Hospital, Marylebone Road, W., £262 10s.

Six Hospitals for Women.—Chelsea Hospital for Women, S.W., £262 10s.; Hospital for Women, Soho Square, W., £367 10s.; Grosvenor Hospital for Women and Children, Vincent Square, S.W., £78 15s.; New Hospital for Women, Euston Road, W.C., £131 5s.; Royal Hospital for Children and Women, Lambeth, S.E., £201 5s.; Samaritan Free Hospital, Lower Seymour Street, W., £490.

### Twenty six other Special Hospitals.

Cancer Hospital, Brompton, S.W., £568 15s.; London Fever Hospital, Islington, N., £288 15s.; Gordon Hospital for Fistula, Vauxhall Bridge Road, S.W., £39 7s. 6d.; St. Mark's Hospital for Fistula, City Road, E.C., £96 5s.; National Hospital for the Diseases of the Heart and Paralysis, Soho Square, W., £87 10s.; Female Lock Hospital, Harrow Road, W., £218 15s.; Male Lock Hospital, Soho, W., £17 10s.; Hospital for Epilepsy, Paralysis, and other Diseases of the Nervous System, Regent's Park, N.W., £52 10s.; National Hospital for the Paralyzed and Epileptic, W.C., £612 10s.; West End Hospital for Diseases of the Nervous System, £35; Central London Ophthalmic Hospital, Gray's Inn Road, W.C., £35; Royal Eye Hospital, St. George's Circus, S.E., £56 17s. 6d.; Royal London Ophthalmic Hospital, Moorfields, E.C., £490; Royal Westminster Ophthalmic Hospital, Charing Cross, W.C., £78 15s.; Western Ophthalmic Hospital, Marylebone Road, W., £14; City Orthopædic Hospital, Hatton Garden, E.C., £61 5s.; National Orthopædic Hospital, Great Portland Street, W., £39 7s. 6d.; Royal Orthopædic Hospital, Oxford Street, W., £70; Hospital for Diseases of the Skin, Stamford Street, S.E., £21 17s. 6d.; St. Peter's Hospital for Stone, Covent Garden, £30 12s. 6d.; Central London Throat and Ear Hospital, Gray's Inn Road, W.C., £39 7s. 6d.; Hospital for Diseases of the Throat, Golden Square, W.C., £35; Royal Ear Hospital, Frith Street, W., £4 7s. 6d.; Phillips Memorial Homœopathic Hospital, Bromley, Kent, £26 5s.; Dental Hospital of London Leicester Square, W.C., £96 5s.; National Dental Hospital, 149, Great Portland Street, W., £30 12s. 6d.

### Twenty-four Convalescent Hospitals.

Metropolitan Convalescent Institution, Sackville Street, £568 15s.; Bexhill Convalescent Home, Sackville Street, £280; All Saints' Convalescent Hospital, Eastbourne, £481 5s.; Mrs. Gladstone's Convalescent Home, Woodford, £113 15s.; Hahnemann Convalescent Home, Bournemouth, £30 12s. 6d.; Hanwell Convalescent Home, £17 10s.; Herbert Convalescent Home, Bournemouth, £30 12s. 6d.; Homœopathic Convalescent Home, Eastbourne, £13 2s. 6d.; King's College Hospital Convalescent Home, Hemel Hempstead, £87 10s.; Mrs. Kitto's Convalescent Home, Reigate, £70; Mrs. Marshman's London and Brighton Female Convalescent Home, £70; Mary Wardell Convalescent Home for Scarlet Fever, £93 5s.; Morley House Convalescent Home for Working Men, £122 10s.; Princess Frederica's Convalescent Home, East Molesey, £35; Boys' Convalescent Home, La Belle Alliance Square, Ramsgate, £17 10s.; St. Andrew's Convalescent Hospital, Clewer, £140; St. Andrew's Convalescent Home, Folkestone, £192 10s.; St. Barnabas Convalescent Home, Ramsgate, £26 5s.; St. John's Home for Convalescent and Crippled Children, Brighton, £26 5s.; St. Joseph's Convalescent Home, Bournemouth, £43 15s.; Convalescent Home for Poor Children, St. Leonard's-on-Sea, £113 15s.; St. Mary Magdalene's Convalescent Home, Paddington, £35; St. Michael's Convalescent Home, Westgate-on-Sea, £39 7s. 6d.; Seaside Convalescent Hospital, Seaford, £140.

### Fourteen Cottage Hospitals.

Beckenham Cottage Hospital, £39 7s. 6d.; Blackheath and Charlton Cottage Hospital, £5[illegible] 17s. 6d.; Bromley, Kent, Cottage Hospital, £56 17s. 6d.; Chertwood, Surrey, Cottage Hospital, £4 7s. 6d.; Chislehurst, Sidcup and Cray Valley Cottage Hospital, £43 15s.; Coldash (Newbury) Convalescent Cottage Hospital, £4 7s. 6d.; Eltham Cottage Hospital, £17 10s.; Enfield Cottage Hospital, £35; Epsom and Ewell Cottage Hospital, £52 10s.; Hounslow Cottage Hospital, £35; Reigate and Redhill Cottage Hospital, £52 10s.; Shedfield Cottage Hospital and Convalescent Home, £4 7s. 6d.; Sidcup Cottage Hospital, £26 5s.; Wimbledon Cottage Hospital, £35.

### Eight Institutions.

Establishment for Gentlewomen, Harley Street, £87 10s.; Hampstead Home Hospital, £78 15s.; National Sanitorium for Consumption, Bournemouth, £5[illegible] 17s. 6d.; Royal Sea Bathing Infirmary, Margate, £437 10s.; Invalid Asylum, Stoke Newington, £52 10s.; Firs Home, Bournemouth, £30 12s. 6d.; St. Catherine's Home, Ventnor, £35; Royal Mineral Water Hospital, Bath, £35.

### Fifty-four Dispensaries.

Battersea Provident Dispensary, £61 5s.; Bloomsbury Provident Dispensary, £18 15s.; Brixton, &c., Dispensary, £48 2s. 6d.; Brompton Provident Dispensary, £21 17s. 6d.; Camberwell Provident Dispensary, £83 2s. 6d.; Camden Provident Dispensary, £9 12s. 6d.; Chelsea, Brompton, and Belgrave Dispensary, £39 7s. 6d.; Child's Hill Provident Dispensary, £3 15s.; City Dispensary, £78 15s.; City of London and East London Dispensary, £21 17s. 6d.; Clapham General and Provident Dispensary, £33 5s.; Eastern Dispensary, £28 17s. 6d.; Farringdon General Dispensary, £39 7s. 6d.; Finsbury Dispensary, £52 10s.; Forest Hill Dispensary, £30 12s. 6d.; Gipsey Hill and Upper Norwood Dispensary, £13 2s. 6d.; Hackney Provident Dispensary, £3 2s. 6d.; Hampstead Provident Dispensary, £39 7s. 6d.; Holloway and North Islington Dispensary, £65 12s. 6d.; Islington Dispensary, £39 7s. 6d.; Kensal Town Provident Dispensary, £10 10s.; Kensington Dispensary, £61 5s.; Kilburn, Maida Vale, and St. John's Wood Dispensary, £39 7s. 6d.; Kilburn Provident Medical Institution, £28; London Dispensary, £17 10s.; London Medical Mission, Endell Street, £56 17s. 6d.; Margaret Street Infirmary for Consumption, £8 15s.; Metropolitan Dispensary, £39 7s. 6d.; Notting Hill Dispensary and Maternity (Provident), £21 17s. 6d.; Paddington Provident Dispensary, £35; Pimlico Provident Dispensary (Lupus Street), £17 10s.; Portland Town Dispensary, £17 10s.; Portobello Road Provident Dispensary, £4 7s. 6d.; Public Dispensary, £43 15s.; Queen Adelaide's Dispensary, £35; Royal General Dispensary, £35; Royal Pimlico Provident Dispensary, £39 7s. 6d.; Royal South London Dispensary, £59 17s. 6d.; St. George's and St. James's Dispensary, £48 2s. 6d.; St. George's, Hanover Square, Provident Dispensary, £43 15s.; St. John's Wood and Portland Town Provident Dispensary, £21 17s. 6d.; St. Marylebone General Dispensary, £33 5s.; St. Pancras and Northern Dispensary, £43 15s.; South Lambeth, Stockwell, and North Brixton Dispensary, £43 15s.; South London Medical Aid Institute, £14; Stamford Hill, Stoke Newington, &c., Dispensary, £43 15s.; Tower Hamlets Dispensary, £39 7s. 6d.; Walworth Provident Dispensary, £7 17s. 6d.; Wandsworth Common Provident Dispensary, £8 15s.; Westbourne Provident Dispensary and Maternity, £13 2s. 6d.; Western Dispensary, £43 15s.; Western General Dispensary, £122 10s.; Westminster General Dispensary, £21 17s. 6d.; Whitechapel Provident Dispensary, £26 5s.

